# Selective Adsorption Separation of Sc(III) and Zr(IV) from Acidic Solutions via Zr-SBA-15 with Rich Oxygen-Vacancy Defective

**DOI:** 10.3390/ma19194108

**Published:** 2026-09-25

**Authors:** Chuanxin Zhang, Lei Shi, Zhonghao Zhang, Xinsheng Li

**Affiliations:** 1 State Key Laboratory of Featured Metal Materials and Life-Cycle Safety for Composite Structures, School of Resources, Environment and Materials, Guangxi University, Nanning 530004, China; 2 Guangxi Key Laboratory of Processing for Non-ferrous Metals and Featured Materials, School of Resources, Environment and Materials, Guangxi University, Nanning 530004, China

**Keywords:** oxygen-vacancy defective, mesoporous molecular sieve, Sc(III)/Zr(IV), adsorption separation

## Abstract

Aiming at the challenging selective separation of zirconium (Zr) and scandium (Sc) in hydrochloric acid systems, this study designed a Zr-SBA-15-30 adsorbent featuring abundant surface oxygen vacancy defects. The as-synthesized material possesses an ordered mesoporous structure, a high specific surface area of 477.606 m^2^/g, and a high density of surface oxygen vacancies. In a strongly acidic binary HCl solution containing both Zr and Sc, Zr-SBA-15-30 exhibited exceptional separation performance, achieving an adsorption capacity of 128 mg/g for Zr and an outstanding separation factor (SF_Sc/Zr_) as high as 3480. Fitting results from adsorption isotherms and kinetic models revealed that the adsorption process is predominantly governed by chemisorption and proceeds mainly via monolayer adsorption. The higher ionic character of the Zr-O bond relative to the Si-O bond, Zr doping induces a redistribution of the local electron cloud density on the framework surface, thereby forming electron-rich centers with higher polarizability and stronger coordination activity. Meanwhile, the oxygen vacancy defects introduced by plasma treatment create defect energy levels within the band gap. This synergistic effect of Zr doping and oxygen vacancies can significantly enhance the separation of Zr/Sc. This work provides a viable material design strategy for the efficient separation of Zr and Sc under strongly acidic conditions.

## 1. Introduction

Scandium (Sc) and zirconium (Zr), as important strategic rare-dispersed metals, possess irreplaceable application value in high-technology fields such as aerospace, new energy, nuclear industry, and electronic communications [1,2,3]. However, the similar ionic radius of Sc(III) and Zr(IV) (0.745 Å and 0.720 Å, respectively) and their similar hydrolysis behavior in aqueous solutions make their separation a challenging issue in the production of high-purity scandium and zirconium [4,5,6]. Especially in the crystalline mother liquor during zirconium production from zircon sand, titanium white waste liquids containing zirconium-bearing titanite ores, zirconium-containing rare earth ores (such as monazite and weathering-crust elution-deposited rare earth ore tailings), and bauxite red mud, some researchers have found that the similar ionic radii and hydrolysis behavior of Sc(III) and Zr(IV) make their separation difficult [7,8,9].

Currently, solvent extraction, chemical precipitation, liquid membrane separation, and adsorption methods have been employed to address the Sc/Zr separation problem. However, these methods generally suffer from limitations such as insufficient separation efficiency, complex process flows, or high economic costs [10,11,12]. Therefore, the development of novel separation materials with high selectivity, high adsorption capacity, and good stability is of great scientific significance and practical value for the efficient utilization of scandium and zirconium resources.

Mesoporous molecular sieve SBA-15, with its highly ordered pore structure, large specific surface area (up to 800–1000 m^2^/g), tunable pore size (5–30 nm), and excellent hydrothermal stability, shows great potential in the adsorption separation field of organic azeotropic [13], Cu/Fe [14], and urease [15]. The incorporation of Zr into the SBA-15 framework yields Zr-SBA-15 composite materials, in which the Zr active sites on the surface can enhance the adsorption capacity for specific metal ions through coordination interactions. Notably, oxygen vacancies (O_Vs_), as common intrinsic defects in metal oxides, can significantly modulate the electronic structure and surface chemical properties of materials [16,17,18,19]. It has been found that oxygen vacancies in Fe_3_O_4_@SBA-15 materials can enhance electron transfer [20,21], and the presence of oxygen vacancies can increase the anchoring sites for Zr on the matrix [22]. In addition, oxygen vacancy defects have also been observed in functionalized SBA-15 materials to reduce the adsorption energy toward adsorbates [23,24]. For example, Zhang et al. found that after the electrochemical introduction of oxygen vacancies, the adsorption capacity of γ-MnO_2_ for UO_2_^2+^ increased from 456.8 mg/g to 1648.1 mg/g, an increase of 3.6 times [25].

Based on the above research background, this study aims to design and prepare oxygen-vacancy-rich Zr-SBA-15 adsorbents and systematically investigate their adsorption and separation performance for Sc(III) and Zr(IV). By introducing oxygen vacancy defects into the Zr-SBA-15 framework, it is expected that the regulatory effect of oxygen vacancies on the coordination environment of metal ions can be utilized to enhance the selectivity toward scandium or zirconium. This study will combine multiple characterization techniques to elucidate the formation of oxygen vacancies and their influence mechanisms on adsorption performance and will explore the microscopic mechanism of oxygen-vacancy-enhanced selective separation of scandium and zirconium through experimental investigations, with the aim of providing new material design strategies and theoretical bases for the efficient separation of scandium and zirconium.

## 2. Experimental

### 2.1. Synthesis of SBA-15, Zr-SBA-15 and Zr-SBA-15 with Rich Oxygen-Vacancy

A beaker containing 120 g of 2 M HCl, 4 g of polyethylene oxide–polypropylene oxide–polyethylene oxide (P123), and 30 g of ultrapure water was stirred at 313 K and 500 rpm for 6 h. Then, 9.14 mL of tetraethyl orthosilicate (TEOS) was added, and the mixture was stirred at 313 K for 24 h, followed by hydrothermal treatment in a Polytetrafluoroethylene (PTFE)-lined autoclave at 373 K for 72 h. The resulting white solid was thoroughly washed with ultrapure water and dried at room temperature for 48 h. Subsequently, it was calcined in a resistance furnace at 823 K for 6 h to obtain SBA-15. In the next step, 1 g of SBA-15 and 50 mL of toluene were placed in a beaker, which was sealed and stirred at 358 K and 300 rpm for 30 min. Then, 1 mL of zirconium n-propanolate was added, and the reflux reaction was maintained under the same conditions for 3 h. The resulting product was washed with ultrapure water three times, suction-filtered, and vacuum-dried at 323 K for 24 h to afford Zr-SBA-15. Finally, the as-prepared SBA-15 and Zr-SBA-15 samples were placed in a dielectric barrier discharge (DBD) plasma reactor and treated at 220 V and 250 W under a 10% O_2_/Ar atmosphere for different durations of 15, 30, 45, and 60 min, yielding adsorbents with varying oxygen vacancy defects, designated as SBA-15-15, SBA-15-30, SBA-15-45, SBA-15-60, Zr-SBA-15-15, Zr-SBA-15-30, Zr-SBA-15-45, and Zr-SBA-15-60.

### 2.2. Surface Characterization of Sorbents

SEM (ZEISS Sigma 360, ZEISS, Oberkochen, Germany) was used to visualize surface morphology and chemical composition. FT-IR (Thermo Scientific Nicolet iS20, Thermo Fisher Scientific, Waltham, MA, USA) revealed the chemical bonds, and shifts in characteristic absorption bands indicated the involvement of functional groups and their subsequent coordination. XRD (Bruker D8 Venture, Bruker, Bremen, Germany) with Cu-Kα radiation (2θ = 10–80°) was employed for crystalline phase analysis. BET (Micromeritics ASAP 2460, Micromeritics Instrument Corporation, Norcross, GA, America) was used to determine pore structure parameters. Electron paramagnetic resonance (EPR) spectroscopy (EMXplus-9.5/12, Bruker, Bremen, Germany) was used to detect unpaired electron centers and calculate g-factors for SBA-15 and Zr-SBA-15 samples with and without plasma treatment. Dried powder samples were loaded into quartz tubes and measured at 298 K, 9.4 GHz, 2 mW, 100 kHz modulation, 0.1 mT amplitude, and a sweep range of 300–400 mT.

### 2.3. Adsorption Experiments

Static batch adsorption experiments were carried out to evaluate the separation performance of the adsorbents and to investigate the effects of HCl concentration, liquid-to-solid ratio, adsorbent dosage, and other parameters. Under a humidity-controlled environment (<20%), a precise amount of adsorbent was weighed using an electronic analytical balance and placed into a 10 mL glass vial, into which the solution was gently added with a micropipette. The vial was securely capped, sealed with parafilm, and then mounted on an orbital shaker inside a benchtop constant-temperature shaking incubator, where it was agitated at 200 rpm at a predetermined temperature. Upon completion of the reaction, the mixture was centrifuged at 4000 rpm for 3 min, and the supernatant was separated from the solid using a 0.45 μm syringe filter. The aqueous phase was diluted with 3 wt% dilute nitric acid at a consistent dilution ratio to an appropriate concentration, and the metal ion concentrations before and after adsorption were determined by inductively coupled plasma optical emission spectroscopy (ICP-OES). All calculations were based on measured values, and each sample solution was analyzed in triplicate to obtain an average. The key parameters used in this adsorption study, namely the sorption efficiency (*E*, %), adsorption capacity (*Q*, mg/g), distribution coefficient (*Kd*, mL/g), and separation factor (), were calculated according to the following equations:

(1) $$E=\frac{\left(C_{0}-C\right)}{C_{0}}\times 100\%$$

(2) $$Q=\frac{\left(C_{0}-C\right)}{m}\times V$$

(3) $$K_{d}=\frac{\left(C_{0}-C\right)}{C}\times \frac{V}{m}\times 1000$$

(4) $${SF}_{A/B}=\frac{K_{dA}}{K_{dB}}$$

where *C*_0_ and *C* are the metal ion concentrations in the aqueous phase before and after adsorption (mg/L), respectively; *V* is the volume of the aqueous phase (mL); *m* is the mass of the adsorbent (mg); and *K_dA_* and *K_dB_* are the distribution ratios of elements A and B, respectively.

## 3. Results and Discussion

### 3.1. Structural Characterization and Analysis of Oxygen Rich Vacancy SBA-15 and Zr-SBA-15 Adsorbents

As illustrated in Figure 1a, the SBA-15 and Zr-SBA-15 samples prepared by hydrothermal method were placed in a dielectric barrier discharge (DBD) plasma reactor. The treatment was carried out at 220 V and 250 W under a 10% O_2_/Ar atmosphere for varying durations of 15, 30, 45, and 60 min. As a result, adsorbents with different oxygen vacancy defects were obtained, which were denoted as SBA-15-15, SBA-15-30, SBA-15-45, SBA-15-60, Zr-SBA-15-15, Zr-SBA-15-30, Zr-SBA-15-45, and Zr-SBA-15-60. Figure 1b,c shows the wide-angle XRD patterns of the SBA-15 and Zr-SBA-15 samples after plasma treatment for different durations. The results indicate that within the scanning range of 2θ = 10–90°, no obvious characteristic diffraction peaks attributable to crystalline silica or zirconia were observed for any of the samples, suggesting that the plasma treatment did not induce long-range ordered rearrangement or crystallization of the framework atoms. It is worth noting that although no significant diffraction information was obtained in the wide-angle region, this does not rule out the existence of the mesostructure in the samples.

Figure 2 presents the N_2_ adsorption–desorption isotherms and the corresponding pore size distribution curves of the SBA-15 and Zr-SBA-15 series samples after plasma treatment for different durations, respectively. According to the IUPAC classification, all samples exhibit typical type IV isotherms with distinct H1-type hysteresis loops (Figure 2a,c), indicating that the plasma treatment did not destroy the inherent open cylindrical mesoporous structure with open ends on both sides (Figure 2b,d), and the mesoscopic orderliness was well preserved [26,27].

The pore structure parameters listed in Table S1 further reveal the differential effects of treatment time on the pore characteristics. For pure silica SBA-15, the specific surface area increased markedly from 565.8 m^2^/g to 660.2 m^2^/g during the initial plasma treatment stage (15 min) and then gradually decreased with extended treatment time (599.1 m^2^/g at 60 min), though it still remained higher than that of the untreated sample. This phenomenon can be ascribed to the dual “cleaning” and “etching” actions of the high-energy active species in the plasma on the pore walls, along with the disruption of the framework Si-O-Si structure that generates oxygen vacancy defects [28,29]. Short-term treatment removes adsorbed impurities from the pores and roughens the surface, thereby exposing a larger specific surface area; however, as the treatment time is further prolonged, extensive cleavage of Si-O-Si bonds may induce localized collapse or shrinkage of the pore walls, resulting in a reduction in surface area. Meanwhile, the pore size of SBA-15 decreased slightly during the early stage of treatment (from 4.03 nm to 3.47 nm), which may be due to the introduction of a new siloxane defect layer on the pore wall surfaces or structural relaxation. In contrast, after plasma treatment, the specific surface area of Zr-SBA-15 remained within a narrow range of 477–497 m^2^/g, and both its pore size (5.88–6.14 nm) and pore volume (0.761–0.802 cm^3^/g) were significantly larger than those of pure silica SBA-15. We speculate that the incorporation of Zr, through the formation of Zr–O-Si bonds, may enhance the framework stability while also modulating the surface electronic structure of the samples [30,31]. Overall, the plasma treatment duration exerts a regulatory effect on the pore structure of both types of materials; nevertheless, the mesoporous structure remains intact and the main pore channels are well preserved.

Figure 3 presents the Fourier-transform infrared (FT-IR) spectra of the Zr-SBA-15 and SBA-15 samples. The absorption peak at around 780 cm^−1^ is attributable to the symmetric stretching vibration of Si-O-Si and Zr-O-Si within the SBA-15 framework. In addition, the absorption band near 1200 cm^−1^ is assigned to the bending vibration of ≡Si-OH (Figure 3a,b), while the broad peak at 3480 cm^−1^ is due to the stretching vibration of -OH groups originating from adsorbed water or silanol groups on the material surface [32]. As the plasma treatment time was prolonged, the intensities of the framework vibration peaks corresponding to Si-O-Si and Zr-O-Si gradually diminished, suggesting that the plasma-induced effects caused the cleavage of some Si-O-Si and Zr-O-Si bonds, which may consequently introduce oxygen vacancy defects into the material framework.

To further investigate the effect of plasma treatment time on the defect structure of the materials, electron paramagnetic resonance (EPR) spectroscopy was employed to characterize the Zr-SBA-15 and SBA-15 series samples at different treatment times (Figure 4). The results show that the pristine samples without plasma treatment exhibited no obvious EPR signals, indicating that there were almost no unpaired electrons in their initial state, i.e., no detectable concentration of oxygen vacancy defects. However, all samples after plasma treatment displayed a single symmetric Lorentzian-type signal with a g-factor of approximately 2.002 [33,34,35]. This characteristic signal can be unequivocally assigned to the paramagnetic center formed by oxygen-vacancy-trapped single electrons, directly confirming that the plasma treatment effectively introduced oxygen vacancy defects into the material framework.

Combined with the FT-IR analysis results, the formation mechanism of oxygen vacancies can be attributed to the bombardment effect of high-energy particles generated by the plasma, which causes the cleavage of Si-O-Si and Zr-O-Si bridging bonds in the framework. Oxygen atoms detach from the lattice in a gaseous form, leaving unpaired electrons at the original oxygen sites, thereby forming oxygen vacancies (V_0_). As the plasma treatment time was extended from 15 min to 60 min, the EPR signal intensity continuously increased, indicating a gradual rise in oxygen vacancy concentration, which demonstrates a significant regulatory effect of treatment time on defect generation. However, when the oxygen-vacancy concentration is too high, the Zr-O-Si or Si-O-Si structures are extensively destroyed (Figure S1), and the Zr-SBA-15 or SBA-15 framework is prone to collapse, which is unfavorable for Sc/Zr adsorption. This is consistent with the adsorption performance results.

It is worth noting that a higher oxygen vacancy concentration is not necessarily more beneficial for material performance. Excessively high defect concentrations may disrupt the integrity of the framework structure, leading to a decrease in pore ordering or even localized structural collapse. This inference is consistent with the observed decreases in specific surface area and degradation of mesoporous structure from the BET specific surface area and pore size distribution data. Based on the combined results of EPR, BET, and IR characterizations, the sample treated with plasma for 30 min maintains a well-preserved mesoporous structure while possessing a moderate oxygen vacancy concentration, exhibiting the optimal balance between structural integrity and defect density. Therefore, the sample prepared under this condition was selected as the optimal material for subsequent adsorption performance tests.

The surface morphologies of the SBA-15-30 and Zr-SBA-15-30 samples after 30 min of plasma treatment were examined by scanning electron microscopy (SEM), and the corresponding micrographs are presented in Figure 5. In comparison with the untreated pristine SBA-15, both plasma-treated samples exhibited surface roughening, with irregular etching scars and slight peeling observed in some localized areas (Figure 5a,b). This morphological evolution suggests that the high-energy active species in the plasma, including ions, free radicals, and excited neutral species, produced a synergistic effect of physical bombardment and chemical etching on the material surface. This etching action not only broke the Si-O-Si and Zr-O-Si bridging bonds in the surface framework layers, causing partial detachment of siloxane tetrahedral units from the framework, but also introduced abundant terminal defects and unsaturated coordination sites on the surface. These observations further support the conclusions regarding framework bond cleavage and oxygen vacancy formation derived from the IR and EPR analyses.

Notably, the Zr-SBA-15-30 sample displayed slightly higher surface roughness than SBA-15-30 (Figure 5c,d), along with a more uniform etching distribution. This may be due to the local framework strain and charge density variations induced by Zr doping, which make the Zr-O-Si bonds more reactive toward plasma species than the Si-O-Si bonds. Despite this, neither sample showed obvious grain fragmentation or overall collapse of the pore structure at the microscopic level, indicating that the 30 min plasma treatment effectively activated the surface while maintaining the overall structural integrity of the materials. This finding is consistent with the BET results, which confirmed that the samples retained a relatively high specific surface area and an ordered mesoporous structure. In conclusion, moderate plasma etching can effectively roughen the surface and increase the exposure of active sites without compromising the main framework structure, which is beneficial for subsequent adsorption processes.

### 3.2. Effect of HCl Solution Concentration on the Adsorption Performance of Sc and Zr

In the scandium extraction process, HCl is commonly used to dissolve minerals, followed by the separation of Sc, Zr, and other substances from the acid solution. Therefore, HCl concentration is an important factor affecting adsorbent performance. Within the HCl concentration range of 1–6 mol/L, the adsorption separation performance of SBA-15, SBA-15-30, Zr-SBA-15, and Zr-SBA-15-30 for Sc(III) and Zr(IV) in a mixed Sc/Zr solution was investigated. The experimental conditions were as follows: liquid-to-solid ratio of 10 mL/10 mg, initial Sc(III) concentration of 1 mmol/L, initial Zr(IV) concentration of 1 mmol/L, temperature of 298 K, adsorption time of 12 h, solution volume of 1 L, adsorbent dosage of 1 g, and shaking speed of 200 rpm.

As shown in Figure 6a,b, for the SBA-15 adsorbent, when the HCl concentration increased from 1 mol/L to 6 mol/L, the sorption efficiency of Zr(IV) exhibited relatively little variation, remaining consistently around 78%. In contrast, the sorption efficiency of Sc(III) showed a continuous decreasing trend with increasing acidity, dropping from 38% to 31% (Figure 6a). The highest separation factor (SF_Sc/Zr_ = 52) was achieved at a concentration of 5 mol/L (Figure 6b). This is mainly attributed to the formation of stable soluble chloro-complexes between Cl^−^ and Sc(III) in the high-concentration HCl system, which significantly reduces the effective concentration of free Sc^3+^ ions in solution, thereby weakening the electrostatic attraction and coordination capture ability of the active sites on the adsorbent surface toward Sc(III). Meanwhile, under concentrated HCl conditions, Zr(IV) and Sc(III) may coexist in the form of multiple chloro-complexes and hydrolyzed species, and their specific distribution depends on experimental conditions such as acidity and Cl^−^ concentration [36].

As shown in Figure 6c,d, after the introduction of oxygen vacancy defects via plasma treatment (SBA-15-30), the acidity dependence of the adsorption system for the two metal ions remained essentially consistent with that of the pristine SBA-15, but the overall adsorption performance was significantly enhanced. The sorption efficiency of SBA-15-30 toward Zr(IV) increased to above 81% under all acidity conditions (Figure 6c). At an HCl concentration of 1 mol/L, the maximum separation factor of the SBA-15-30 system reached 72 (Figure 6d), representing an improvement of approximately 38.5% over that of SBA-15. The main reasons for this are as follows. On the one hand, plasma etching roughened the material surface and increased the number of unsaturated coordination sites (such as Si-O· and oxygen vacancy defects), providing more chelating adsorption centers for Zr(IV). On the other hand, the introduction of oxygen vacancies altered the local charge distribution on the framework surface, enhancing the selective affinity toward the highly charged Zr^4+^ ions while suppressing the affinity for Sc^3+^, thereby further amplifying the difference in adsorption affinity between the two ions.

In the Zr-SBA-15 adsorption system (Figure 7a,b), as the HCl concentration increased from 1 mol/L to 6 mol/L, the sorption efficiency of Zr(IV) remained highly stable, consistently maintained at approximately 92%. This indicates that the Zr-doped framework structure possesses exceptionally strong affinity for Zr(IV) and excellent resistance to acid interference. The increases in H^+^ concentration and Cl^−^ coordination competition in the solution had almost no effect on its capture efficiency. In contrast, the sorption efficiency of Sc(III) exhibited a significant decreasing trend with increasing acidity, dropping from 24% to 12%. This is attributed to the transformation of Sc(III) into soluble chloro-complexes in the high-concentration Cl^−^ system, which greatly reduces the activity of free Sc^3+^ ions, thereby weakening the adsorption of Sc(III) on the adsorbent surface [37]. It is precisely the presence of such soluble chloro-complexes (e.g., ScCl_4_^−^, ScCl_6_^3−^ and ScCl_3_) that reduces the amount adsorbed, thereby increasing the separation factor SF_Sc/Zr_. This indicates that Zr doping may be attributed to modulating the electronic structure of surface active sites and enhancing the selective adsorption toward Zr(IV).

In the Zr-SBA-15-30 adsorption system (Figure 7c,d), after the introduction of oxygen vacancy defects via plasma treatment, the adsorption performance was further enhanced. The adsorption rate of Zr(IV) exceeded 98% under all acidity conditions, while the sorption efficiency of Sc(III) was significantly suppressed to extremely low levels (Figure 7c). On this basis, the maximum separation factor of the system reached as high as 3480 at an HCl concentration of 4 mol/L, representing a 35-fold improvement over that of Zr-SBA-15 (Figure 7d). This excellent separation performance can be attributed to the synergistic enhancement mechanism between Zr doping and oxygen vacancy defects. On the one hand, the incorporation of Zr into the framework forms Zr-O-Si bonds, whose local charge density is higher than that of the original Si-O-Si units [36]. This induces charge redistribution on the framework surface and enhances the electrostatic affinity of the adsorption sites toward the highly charged Zr^4+^ ions. On the other hand, the oxygen vacancy defects generated by plasma bombardment further increase the number of unsaturated coordination sites on the surface and introduce electron-rich paramagnetic centers (g ≈ 2.002), which confer stronger coordination chelating capability toward Zr^4+^. The synergy between these two effects manifests as follows: the Zr-O-Si framework provides the intrinsic basis for selectivity, while the oxygen vacancy defects greatly amplify the preferential adsorption advantage toward Zr(IV) on this basis. Notably, this synergistic effect does not simultaneously enhance the adsorption of Sc(III); instead, Sc(III) is further suppressed due to its weaker surface complexation ability and its tendency to form stable chloro-complexes under high acidity. This “enhancement–suppression” coupling mechanism enables Zr-SBA-15-30 to maintain an exceptionally high separation factor over a wide acidity range (especially at 4 mol/L HCl), far outperforming the control samples without Zr doping or plasma treatment.

To optimize adsorbent utilization efficiency and maximize the separation performance between Sc(III) and Zr(IV), the effect of the liquid-to-solid ratio (i.e., adsorbent dosage) on selective adsorption behavior was investigated. The experimental conditions were as follows: solution volume of 10 mL, initial concentrations of both Sc(III) and Zr(IV) at 1 mmol/L, HCl concentration of 4 mol/L, temperature of 298 K, adsorption time of 12 h, and shaking speed of 200 rpm; the liquid-to-solid ratio was varied by adjusting the adsorbent dosage.

As shown in Figure 8a, with increasing Zr-SBA-15-30 dosage, the sorption efficiency of Zr(IV) exhibited a continuous upward trend, while its unit-mass adsorption capacity (Q) gradually decreased. This typical variation pattern can be attributed to the relative balance between the number of adsorption sites and the total amount of metal ions in solution. At a low adsorbent dosage (<10 mg), the amount of Zr(IV) ions carried per unit mass of adsorbent is relatively high, and the adsorption sites tend to become saturated; therefore, the adsorption capacity remains at a relatively high level, but the residual Zr(IV) concentration in solution is still non-negligible, resulting in a relatively low adsorption rate. As the adsorbent dosage increases, the total number of available active sites correspondingly increases, allowing Zr(IV) in solution to be more thoroughly adsorbed, and the sorption efficiency is significantly improved.

When the adsorbent dosage exceeded 10 mg, the sorption efficiency of Zr(IV) remained constant, indicating that Zr(IV) in the solution had been essentially completely adsorbed. Under these conditions, however, the sorption efficiency of Sc(III) continued to increase with further increases in adsorbent dosage. This phenomenon is mainly attributable to the significant difference in the adsorption behavior of Zr-SBA-15-30 toward the two metal ions: the surface of Zr-SBA-15-30 exhibits extremely strong preferential selectivity for Zr(IV), and its active sites are first preferentially occupied by Zr(IV). Only after the Zr-SBA-15-30 dosage is increased to a level sufficient to completely bind all Zr(IV) in solution do the excess active sites begin to bind Sc(III), which has a relatively weaker affinity, leading to an increase in the sorption efficiency of Sc(III). Therefore, the optimal adsorbent dosage is 10 mg, at which the sorption efficiency of Zr(IV) reaches a saturated level (>99%), while the co-adsorption of Sc(III) remains at a relatively low level. This enables efficient recovery of Zr(IV) and maximum retention of Sc(III), effectively avoiding the loss of Sc in the separation system.

As shown in Figure 8b, as the Sc/Zr molar ratio decreased (i.e., as the Zr(IV) concentration in solution gradually increased), the adsorption behavior of Zr-SBA-15-30 toward Zr(IV) exhibited significant nonlinear characteristics. When the Sc/Zr molar ratio was decreased from 4:1 to 1:1, the sorption efficiency of Zr(IV) remained at a relatively high level, indicating that within this concentration range, the number of active sites on the adsorbent surface was sufficient to effectively bind the Zr(IV) ions in solution, and the strong affinity advantage of the material toward Zr(IV) was fully manifested. However, when the Sc/Zr molar ratio further decreased below 1:1 (i.e., when the Zr(IV) concentration exceeded the Sc(III) concentration), the sorption efficiency of Zr(IV) showed a sharp decline. The initial concentrations of both Zr and Sc were 2000 mg/L. When the solid–liquid ratio was 1:1, the equilibrium concentration of Zr was 40 mg/L, and that of Sc was 1940 mg/L. At adsorption equilibrium, the *K_Zr_* and *K_Sc_* were 980 and 0.28. The separation factor of Zr/Sc reached 3480. The main reason for this is that the unit-mass adsorption capacity (Q) of Zr-SBA-15-30 for Zr(IV) had reached its saturation limit; the surface oxygen vacancy defect sites and the Zr-O-Si framework active centers had been completely occupied by Zr(IV). Even as the Zr(IV) concentration in solution continued to increase, the adsorbent was unable to adsorb additional Zr(IV), resulting in excess Zr(IV) remaining in the liquid phase and a sharp drop in the adsorption efficiency. Of course, real acidic leachates typically contain multiple competing metal ions, and ions such as Fe(III), Al(III), Ti(IV), and other rare-earth elements can affect the Zr/Sc separation [38,39]. Based on the hard–soft acid–base (HSAB) theory and the affinity of our functional groups, due to differences in ionic radius, charge density, and coordination geometry, Zr-SBA-15-30 exhibits a higher affinity for Zr than for Fe(III), Al(III), and Ti(IV). However, the presence of these ions can also reduce the Zr/Sc separation factor to some extent.

### 3.3. Adsorption Mechanism

The adsorption kinetics of Zr-SBA-15-30 for Sc(III) and Zr(IV) were examined in single-element HCl solutions. The experiments were conducted under the following conditions: liquid-to-solid ratio of 10 mL/10 mg, initial metal ion concentration of 300 mg/L, HCl concentration of 4 mol/L, shaking speed of 200 rpm, and temperature of 298 K. The residual concentrations of metal ions were measured at various time points after adsorption. In our previous work on a similar system, we conducted a detailed study on the sorption mechanism of the DPO/SBA-15-N adsorbent without oxygen vacancy defects for the separation of Zr/Sc [40]. Comparing the Langmuir and Freundlich adsorption isotherm models, we found that the Langmuir adsorption mechanism applies to mesoporous materials. Zr-SBA-15 is also a mesoporous material; we directly adopted the Langmuir adsorption mechanism for a detailed analysis. The Langmuir adsorption isotherm model was employed to fit the experimental data in order to quantify the maximum adsorption capacity of the adsorbent for the two metal ions and the distribution characteristics of surface active sites. The fitted parameters are listed in Table S4.

As illustrated in Figure 9a, Zr-SBA-15-30 demonstrated extremely fast adsorption kinetics toward Zr(IV), with adsorption equilibrium achieved within 40 min. In the single-element Sc(III) solution, the adsorption capacity increased rapidly during the first 60 min and reached 4 mg/g at an adsorption time of 90 min (Figure 9b). The pseudo-second-order kinetic fitting curves for Sc(III) and Zr(IV) are shown, and the key parameters of the adsorption kinetic models are summarized in Table S2. The correlation coefficient (R^2^) of the pseudo-second-order kinetic model for the adsorption of both Zr(IV) and Sc(III) onto Zr-SBA-15-30 was 0.998, and the theoretical adsorption capacity was in good agreement with the experimental value. This indicates that the adsorption of Zr(IV) and Sc(III) by the adsorbent follows the pseudo-second-order kinetic model more closely, suggesting that the adsorption process may be related to chemisorption. The fitting results of isotherm models only reflect the macroscopic adsorption equilibrium behavior and that their mechanistic interpretation still requires comprehensive judgment in combination with other evidence.

At 298 K, when the initial Zr concentration ranged from 0 to 400 mg/L, the adsorption capacity of Zr-SBA-15-30 for Zr increased rapidly with increasing initial Zr concentration. After the initial concentration exceeded 60 mg/L, the adsorption capacity reached a plateau, with a maximum value of 128 mg/g (Figure 10a). However, Zr-SBA-15-30 showed almost no adsorption toward Sc, with an adsorption capacity of only 4 mg/g (Figure 10b). Within the studied concentration range, the Langmuir model gives the best fit to the experimental data (Table S4), indicating that the adsorption behavior may be described by a monolayer adsorption model onto Zr-SBA-15-30.

Based on the comprehensive characterization results and adsorption performance data, the significant selective adsorption difference of Zr-SBA-15-30 toward Zr(IV) and Sc(III) may be attributed to the synergistic regulatory effect of oxygen vacancy defects and Zr doping on the surface electronic structure of the framework (as illustrated in Figure 11). The proposed mechanism is as follows:

Upon incorporation of Zr into the SBA-15 framework, Zr-O-Si bridging bonds replace a portion of the Si-O-Si units. Owing to the lower electronegativity of Zr^4+^ compared with Si^4+^ and the higher ionic character of the Zr-O bond relative to the Si-O bond, Zr doping induces a redistribution of the local electron cloud density on the framework surface, forming electron-rich centers with higher polarizability and stronger coordination activity. Meanwhile, the oxygen vacancy defects introduced by plasma treatment create defect energy levels within the band gap. The unpaired electrons trapped at these defect sites (g ≈ 2.003, as verified by EPR) endow the adsorption sites with unsaturated coordination capability and additional electron-donating ability. We speculate that the synergy of these two effects may give rise to a unique “electron-rich and coordination-deficient” electronic configuration on the surface active sites, thereby exhibiting selective adsorption characteristics toward Zr^4+^ ions with high charge density and strong polarizing ability.

In the HCl medium, the existing forms of Zr(IV) and Sc(III) and their interaction modes with the adsorption sites are fundamentally different. Zr^4+^, with its high charge density and strong hydrolysis coordination ability, predominantly exists in solution as oxygenated cationic species such as ZrO^2+^ and [Zr(OH)_2_]^2+^. These cationic species exhibit strong electrostatic attraction and coordination chelating ability toward the oxygen vacancy defect sites (i.e., unsaturated Si-O and Zr-O dangling bonds) [41]. The unsaturated O atoms exposed at the oxygen vacancies can act as electron donors, forming stable coordination bonds with the empty orbitals of Zr(IV), thereby achieving effective chemisorption. In addition, the Zr-O-Si framework itself exhibits a “homologous affinity” effect toward Zr(IV), which confers lower steric hindrance and higher bond stability for the binding of Zr(IV) at the adsorption sites.

## 4. Conclusions

In this work, a Zr-SBA-15-30 adsorbent with abundant oxygen vacancy defects on the surface was designed to address the selective separation of Zr and Sc in HCl solution. The adsorbent possesses an ordered mesoporous structure, a high specific surface area of 477.606 m^2^/g, and abundant oxygen vacancy defects. In a strongly acidic HCl binary solution containing Zr and Sc, Zr-SBA-15-30 exhibited excellent separation performance, with a separation factor (SF_Sc/Zr_) as high as 3480 and an adsorption capacity toward Zr reaching 128 mg/g. Owing to the lower electronegativity of Zr^4+^ compared with Si^4+^ and the higher ionic character of the Zr-O bond relative to the Si-O bond, Zr doping induces a redistribution of the local electron cloud density on the framework surface, thereby forming electron-rich centers with higher polarizability and stronger coordination activity. Meanwhile, the oxygen vacancy defects introduced by plasma treatment create defect energy levels within the band gap. This synergistic effect of Zr doping and oxygen vacancies can significantly enhance the separation of Zr/Sc. This work provides a viable material design strategy for the efficient separation of Zr and Sc under strongly acidic conditions.

## Figures and Tables

**Figure 1:**
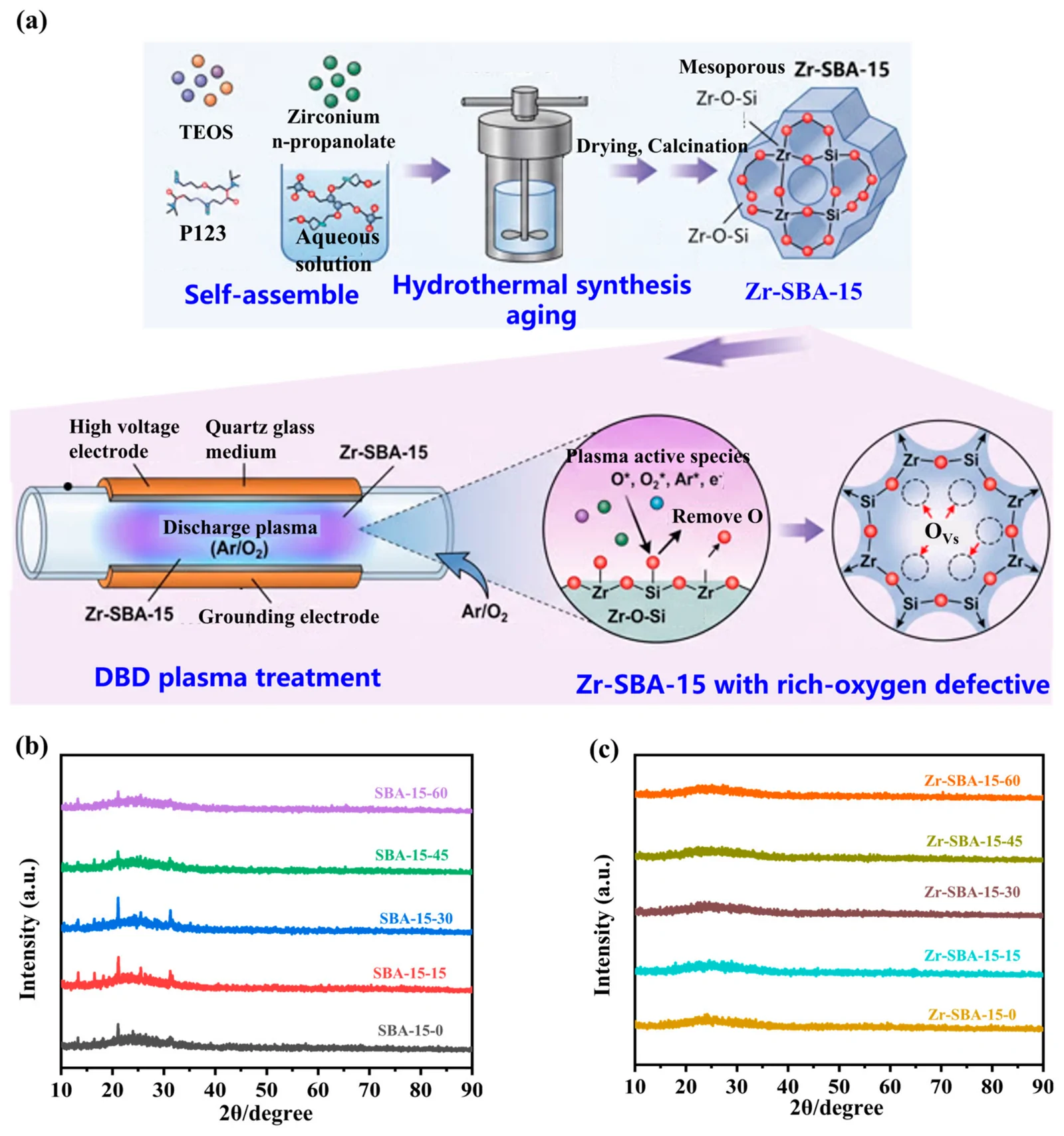
(**a**) Schematic diagram of preparation of oxygen rich vacancy defect Zr-SBA-15. XRD patterns of SBA-15 (**b**) and Zr-SBA-15 (**c**) at different plasma treatment times.

**Figure 2:**
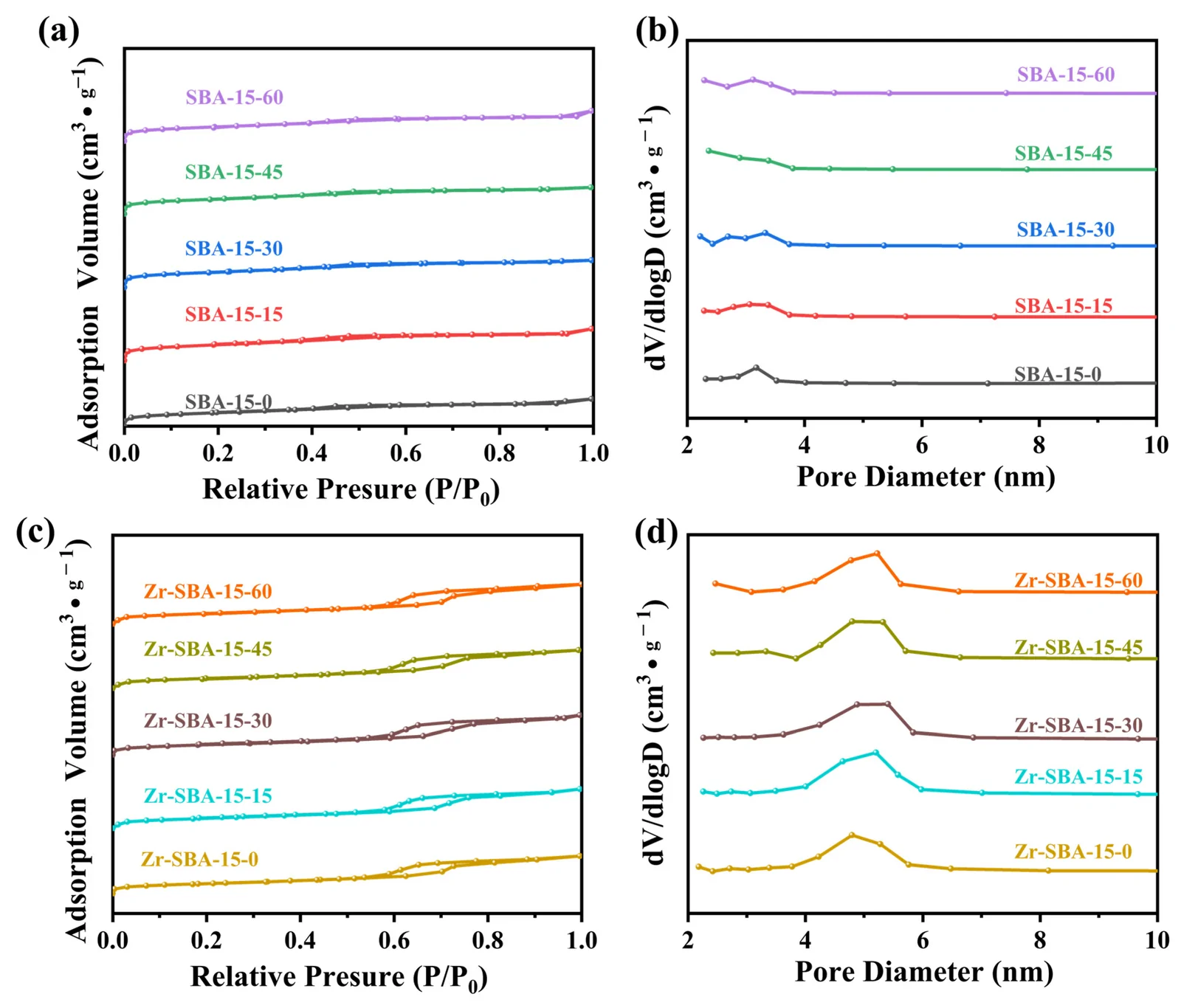
N_2_ isothermal adsorption–desorption curves and pore size distribution of SBA-15 (**a**,**b**) and Zr-SBA-15 (**c**,**d**) under different plasma treatment times.

**Figure 3:**
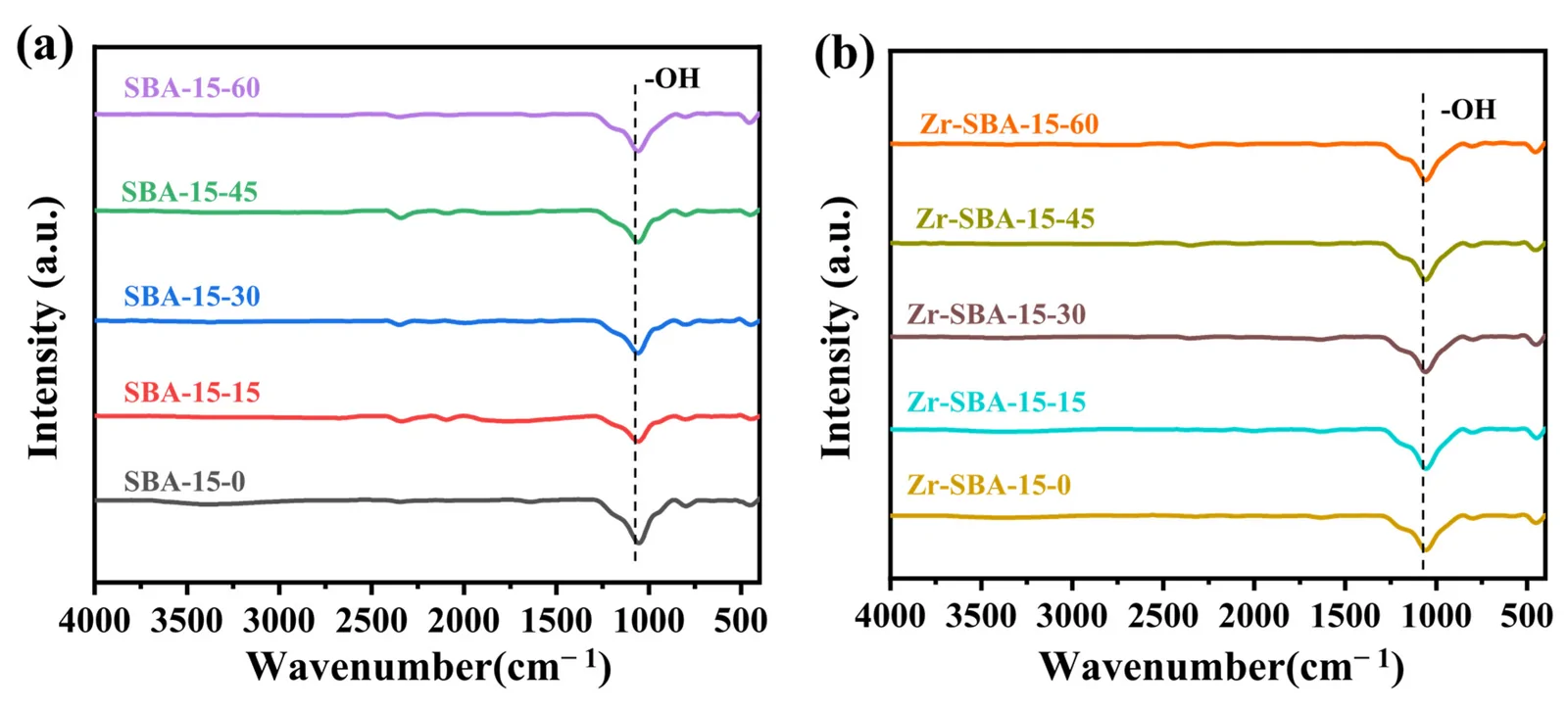
IR spectra of SBA-15 (**a**) and Zr-SBA-15 (**b**) at different processing times.

**Figure 4:**
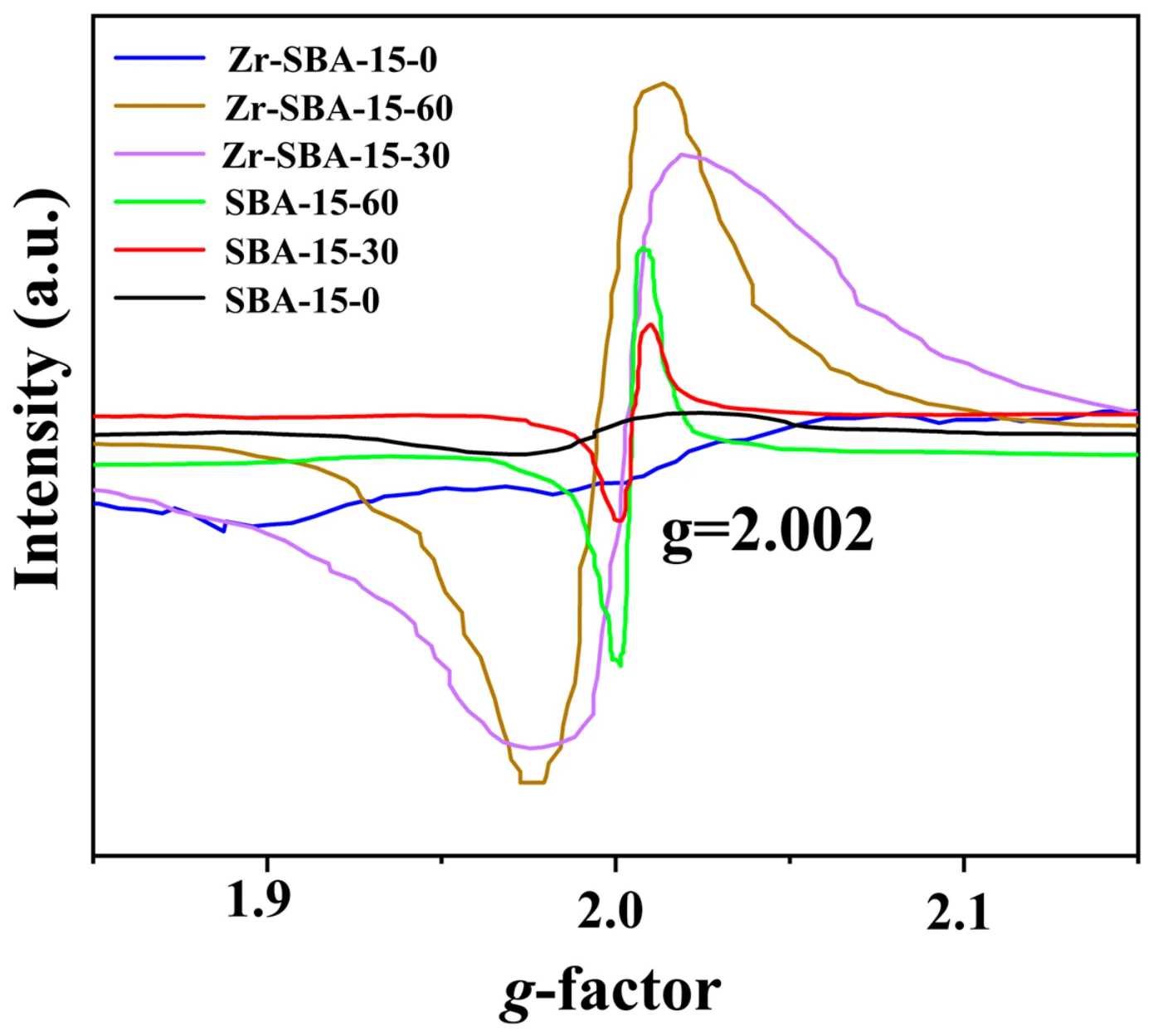
The EPR of SBA-15 and Zr-SBA-15 with different plasma treatment times.

**Figure 5:**
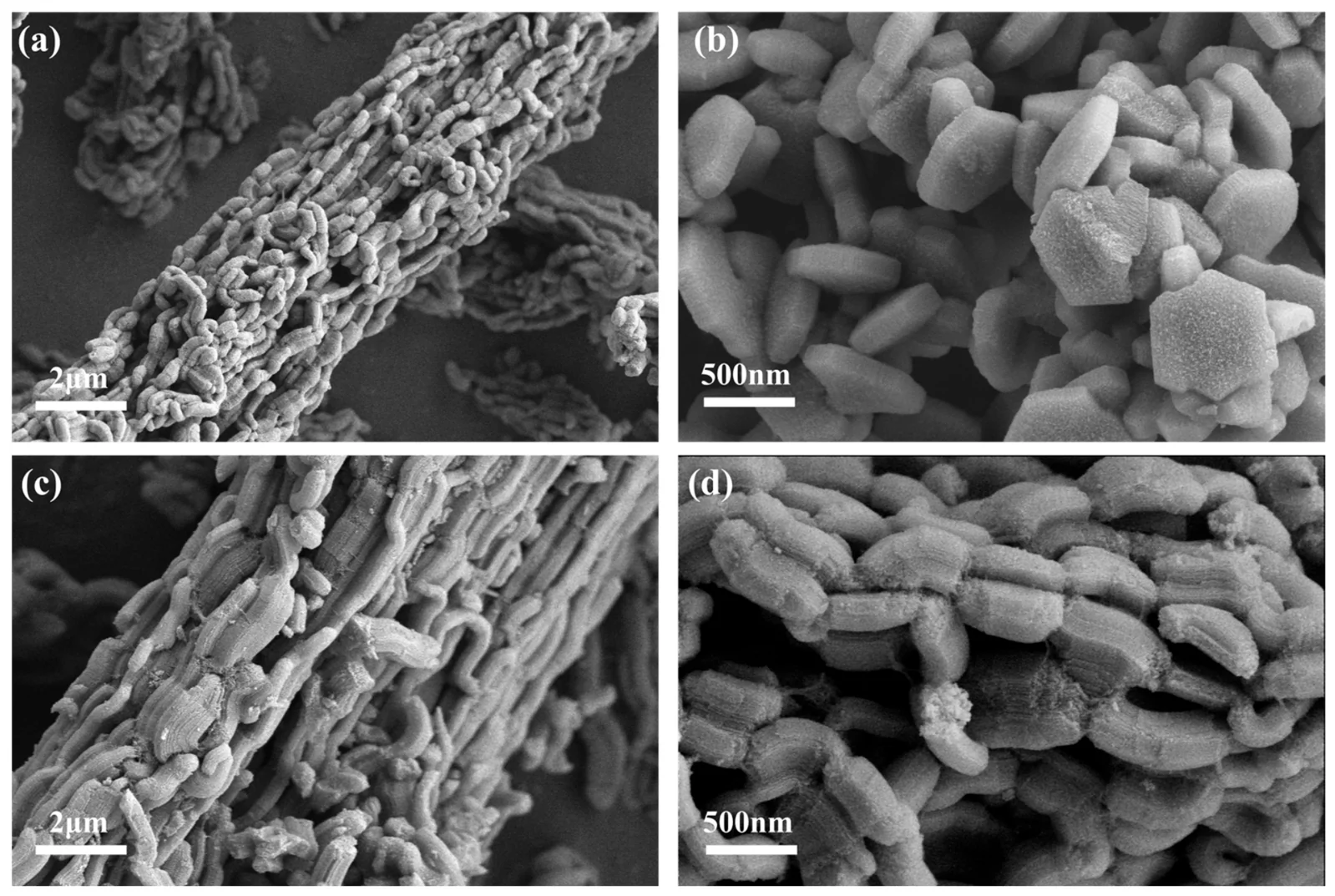
SEM of (**a**,**b**) SBA-15-30 and (**c**,**d**) Zr-SBA-15-30.

**Figure 6:**
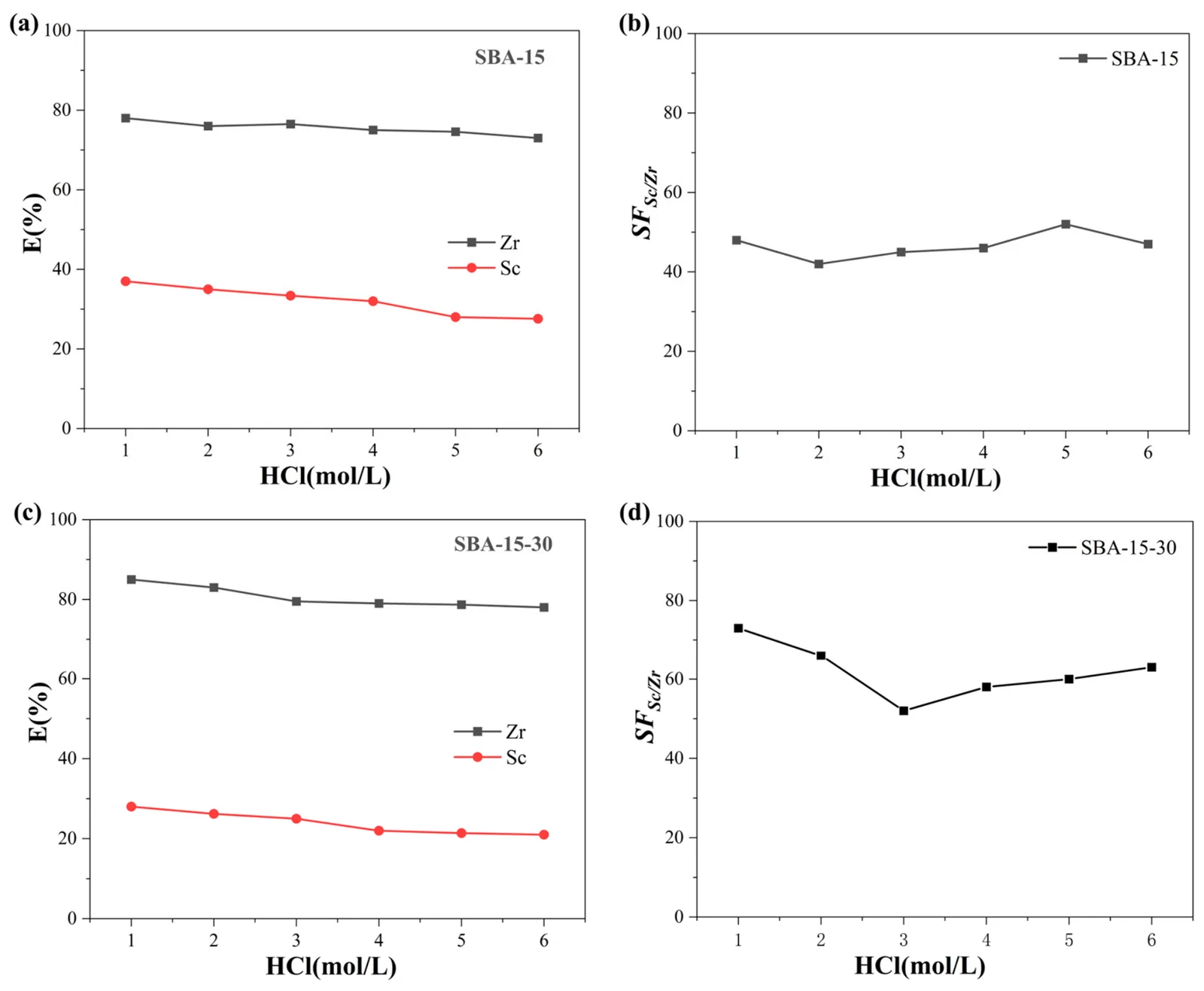
Effect of HCl concentration on the adsorption separation performance of Sc(III)-Zr(IV) on SBA-15 (**a**,**b**) and SBA-15-30 (**c**,**d**).

**Figure 7:**
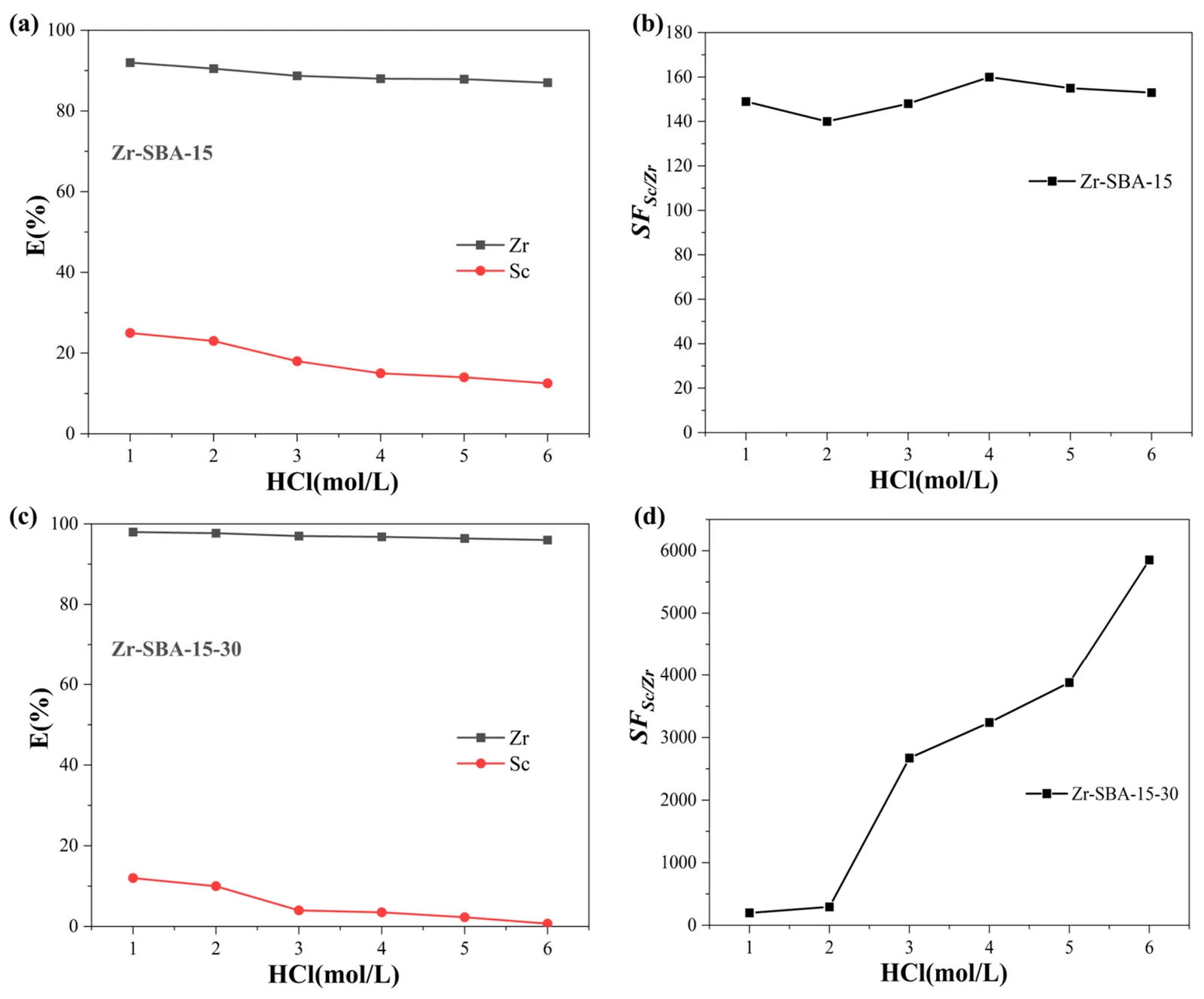
Effect of HCl concentration on the adsorption separation performance of Sc(III)-Zr(IV) on Zr-SBA-15 (**a**,**b**) and Zr-SBA-15-30 (**c**,**d**).

**Figure 8:**
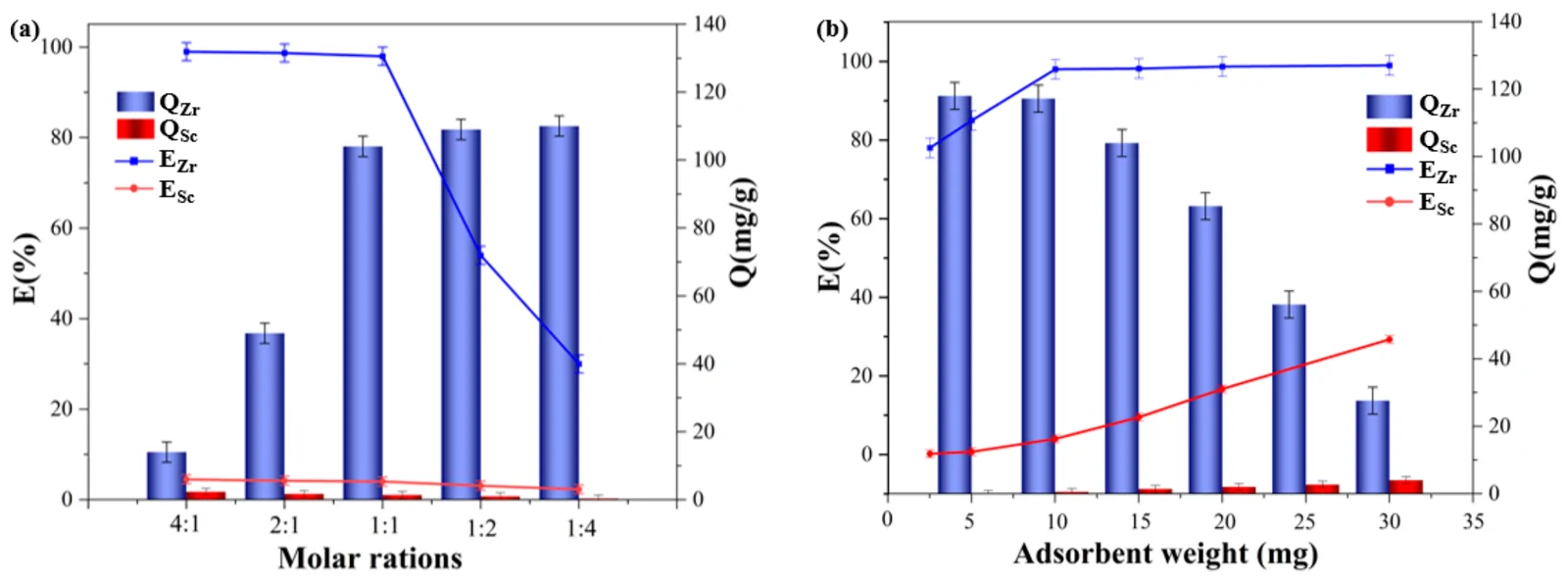
(**a**) Effect of Zr-SBA-15-30 adsorbent dosage on the separation of Sc(III) and Zr(IV). (**b**) Effect of Sc(III) and Zr(IV) molar rations on the adsorption and separation performance of Zr-SBA-15-30.

**Figure 9:**
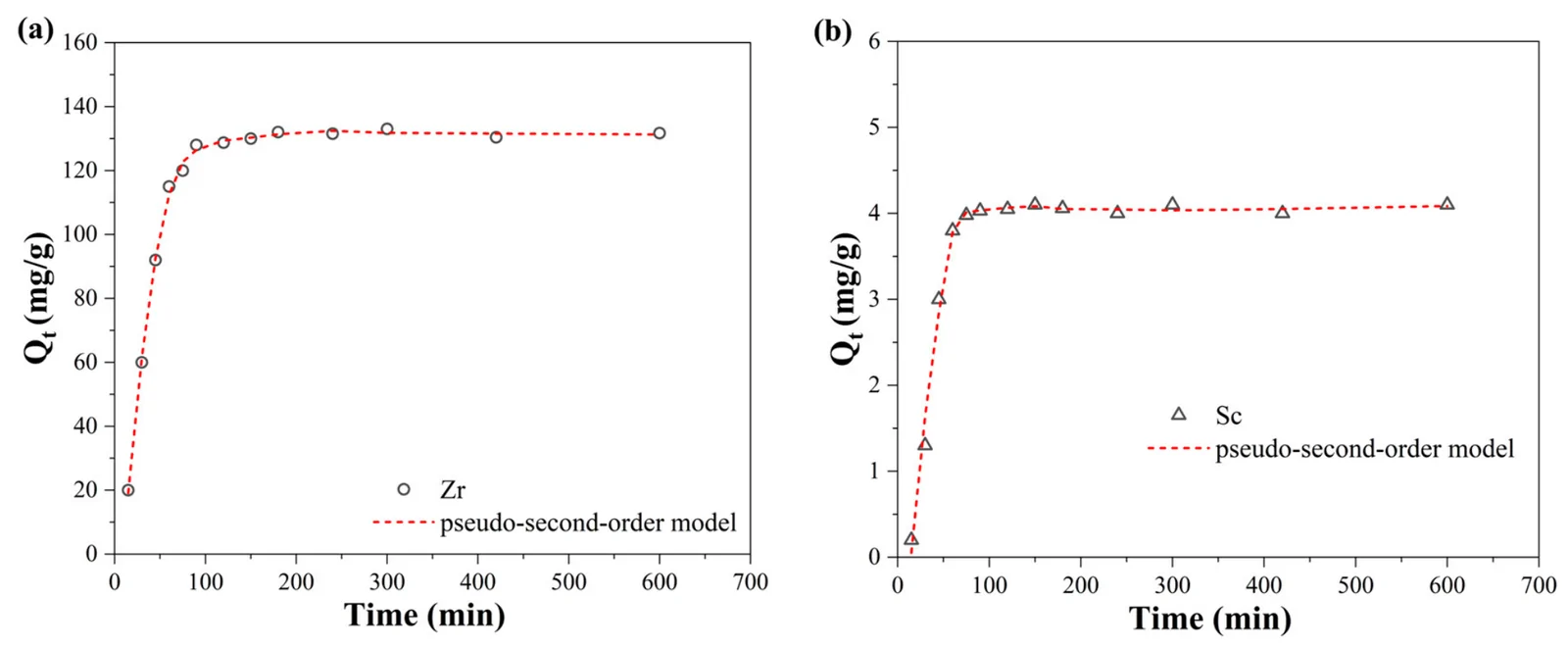
(**a**) adsorption kinetics model of Zr (IV) by Zr-SBA-15-30. (**b**) adsorption kinetics model of Sc (III) by Zr-SBA-15-30.

**Figure 10:**
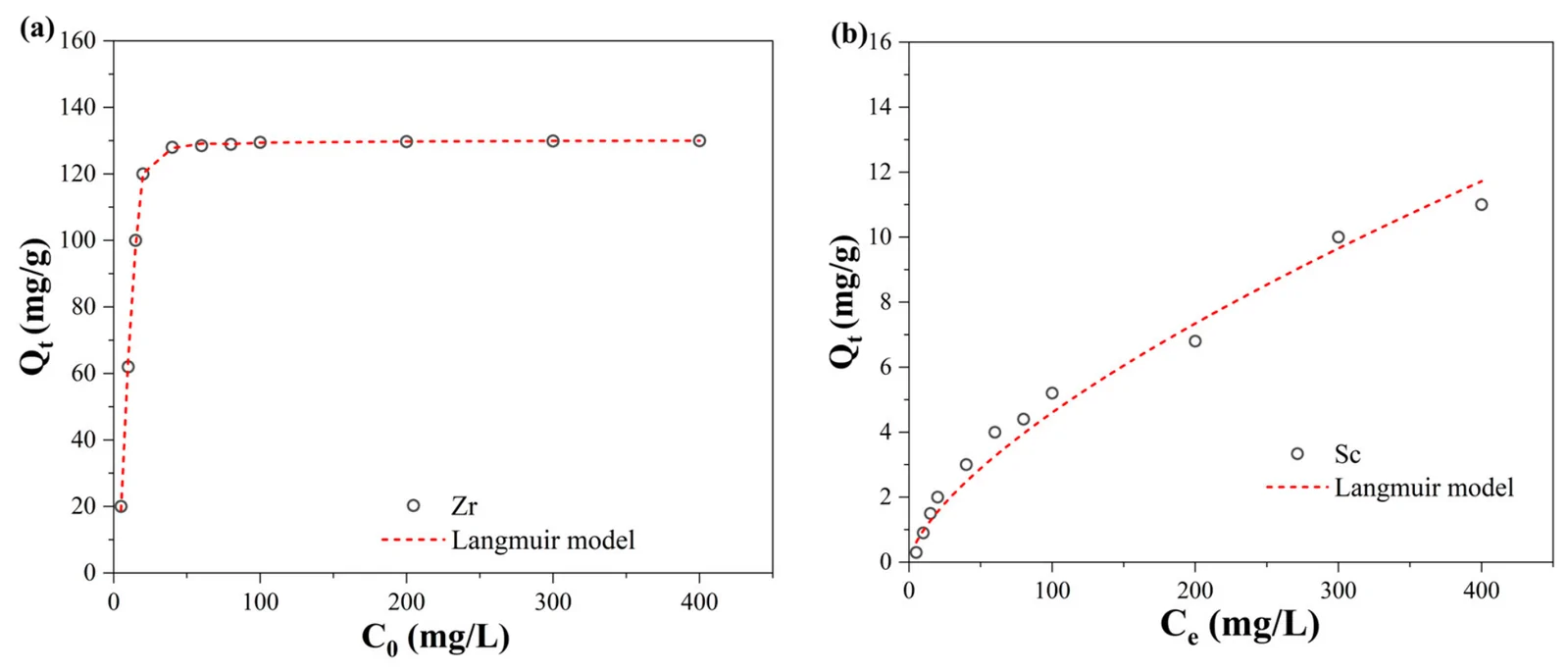
(**a**) adsorption isotherm model of Zr-SBA-15-30 for Zr (IV). (**b**) adsorption isotherm model of Zr-SBA-15-30 for Sc (III).

**Figure 11:**
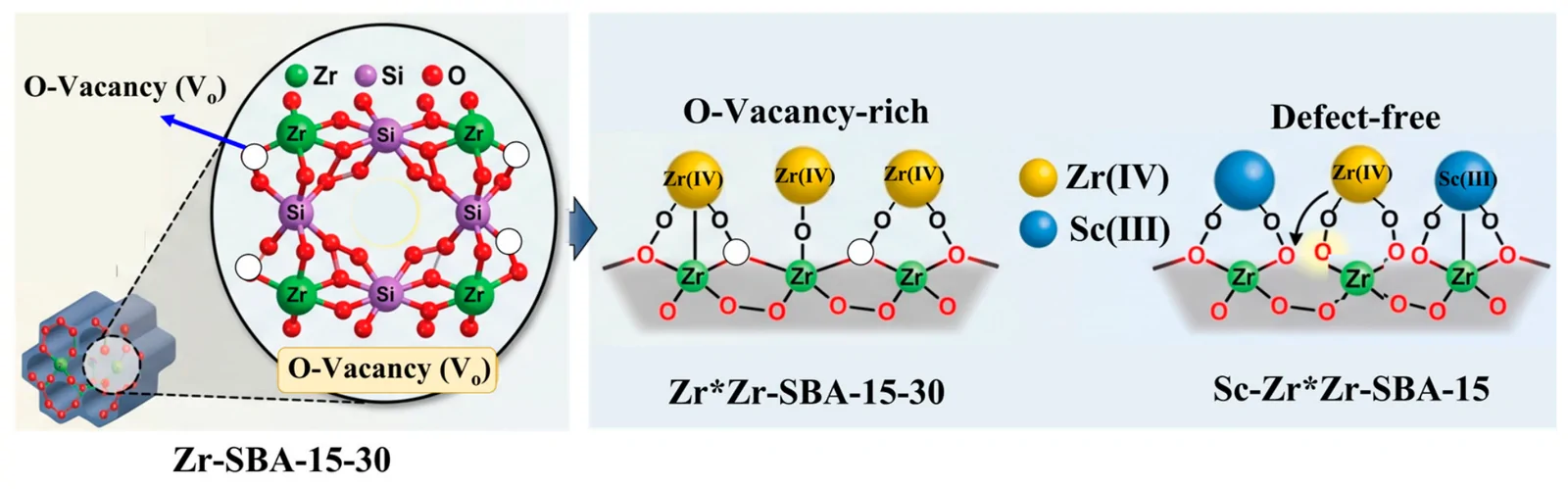
Selective adsorption mechanism of Zr-SBA-15-30 for Sc(III) and Zr(IV).* is the adsorption site.

## Data Availability

The original contributions presented in this study are included in the article/Supplementary Materials. Further inquiries can be directed to the corresponding author.

## Supplementary Materials

The following supporting information can be downloaded at: https://www.mdpi.com/article/10.3390/ma19194108/s1, Figure S1: The mass concentration of Zr-SBA-15 and Zr-SBA treated by plasma with different time. Figure S2: FT-IR spectra of Zr-SBA-15-30 and Zr-SBA-15-30 after Zr adsorption. Table S1: Pore structure information of SBA-15 and Zr-SBA-15 samples. Table S2: The experimental conditions of Zr/Sc separation selectivity. Table S3: Kinetic fitting related parameters of Sc and Zr at 298 K. Table S4: Parameters of adsorption isotherm model for Sc and Zr at 298 K. Table S5: Compared with previously reported adsorbents or separation systems under comparable conditions. References [1,40] are cited in the Supplementary Materials.
