# Supplementary material for: Selective Adsorption Separation of Sc(III) and Zr(IV) from Acidic Solutions via Zr-SBA-15 with Rich Oxygen-Vacancy Defective
Source: Materials (Basel). 2026 Sep 25;19(19):4108. doi: 10.3390/ma19194108 (PMC13665965; doi:10.3390/ma19194108)
Supplement: Supplementary file 1 [file materials-19-04108-s001.zip › materials-4565567-supplementary.pdf]

Supplementary material

Selective adsorption separation of Sc(III) and Zr(IV) from acidic solutions via Zr-SBA-15 with rich oxygen-vacancy defective

Chuanxin Zhang, Lei Shi, Zhonghao Zhang and Xinsheng Li *

State Key Laboratory of Featured Metal Materials and Life-cycle Safety for Composite Structures, Guangxi Key Laboratory of Processing for Non-ferrous Metals and Featured Materials, School of Resources, Environment and Materials, Guangxi University, Nanning 530004, China

* Correspondence: Xinsheng Li (A/Prof.) xinshengli@gxu.edu.cn (X.L.)

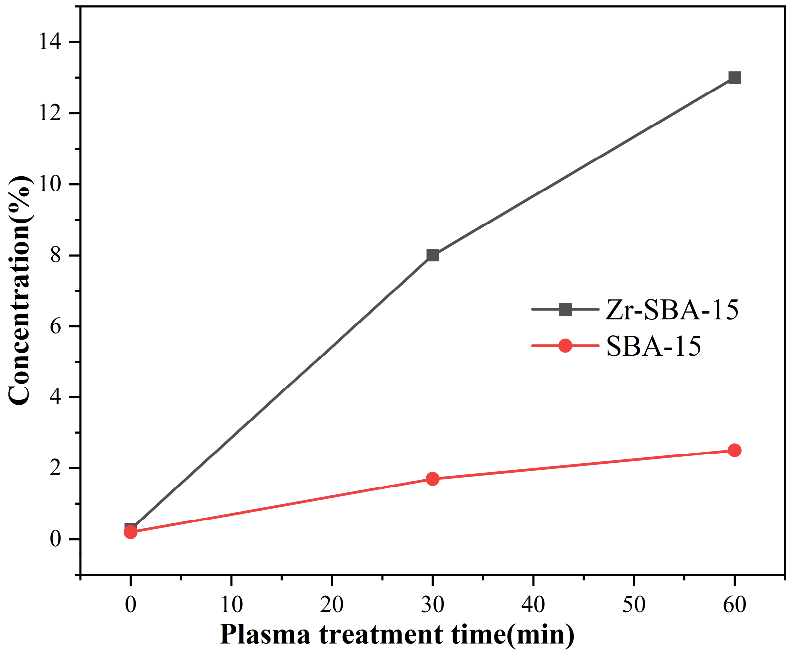

Fig. S1 The mass concentration of Zr-SBA-15 and Zr-SBA treated by plasma with different time.

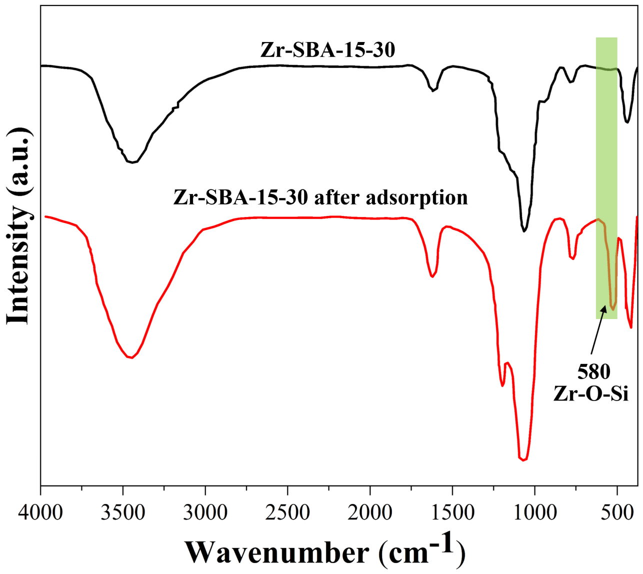

Fig. S2 FT-IR spectra of Zr-SBA-15-30 and Zr-SBA-15-30 after Zr adsorption.

Table S1 Pore structure information of SBA-15 and Zr-SBA-15 samples.

**Sample**	**BET surface area (m^2/g)**	**BJH pore size (nm)**	**Pore volume (cm^3/g)**
SBA-15-0	565.835	4.026	0.494
SBA-15-15	660.215	3.872	0.571
SBA-15-30	650.592	3.468	0.499
SBA-15-45	633.015	3.482	0.485
SBA-15-60	599.054	4.063	0.536
Zr-SBA-15-0	481.304	5.880	0.761
Zr-SBA-15-15	483.203	5.952	0.796
Zr-SBA-15-30	477.606	6.136	0.794
Zr-SBA-15-45	476.416	5.909	0.779
Zr-SBA-15-60	497.314	6.011	0.802

Table S2 The experimental conditions of Zr/Sc separation selectivity

Initial concentrations of Zr and Sc	Zr: 1 mmol/L; Sc: 1 mmol/L
Adsorbent dosage	1 g
Solution volume	1 L
HCl concentration	1-6 mol/L
Contact time	12 h
Temperature	298 K
Liquid-solid- ratio	10 mL/10 mg
Final equilibrium pH	At all HCl concentrations, the initial pH was almost equal to the pH at adsorption equilibrium.

Table S3 Kinetic fitting related parameters of Sc and Zr at 298 K

Element	Pseudo-second-order		
	K (g/(mg·min))	Q_e (mg/g)	R^2
Sc	0.602	4	0.996
Zr	0.104	128	0.998

Table S4 Parameters of adsorption isotherm model for Sc and Zr at 298 K

Element	Freundlich model		
	K_F ((mg^{1-n}·L^n)/g)	n	R^2
Sc	0.079	1.54	0.907
Zr	61.823	7.24	0.861

Table S5 Compared with previously reported adsorbents or separation systems under comparable conditions.

Adsorbents	Acid concentration	$SF_{Zr/Sc}$	Ref.
DPO/SBA-15-N	HCl: 4 mol/L	60	R1
	HCl: 5.5 mol/L	120	
	HNO_3: 6 mol/L	6500	
SiAcP	HCl: 0.5 mol/L	48	R2
	HNO_3: 0.5 mol/L	140	
SBA-15	HCl: 5 mol/L	55	This work
SBA-15-30	HCl: 1 mol/L	72	This work
Zr-SBA-15	HCl: 4 mol/L	163	This work
Zr-SBA-15-30	HCl: 4 mol/L	3480	This work

[R1] Zhonghao Zhang, Xinsheng Li, Sirun Qin, Xiwen Liao, Xiangxi Huang, Duoqiang Liang. Selective adsorption of zirconium from scandium by novel diphosphoryl-modified SBA-15 adsorbent, Journal of Rare Earths, 2025, 44(4):1254-1264. DOI:10.1016/j.jre.2025.04.004.

[R2] Wei Zhang, Shunyan Ning, Shichang Zhang, Siyi Wang, Jie Zhou, Xinpeng Wang, Yuezhou Wei, Synthesis of functional silica composite resin for the selective separation of zirconium from scandium, Microporous and Mesoporous Materials, 2019, 288(c):109602. DOI:10.1016/j.micromeso.2019.109602.
